# Programmable enhancement of endogenous mRNA translation through CRISPR-guided epitranscriptomic regulation

**DOI:** 10.1038/s41421-026-00903-7

**Published:** 2026-06-24

**Authors:** Yaxian Cheng, Guanglin Zhu, Xinzhi Zhou, Yiran Liu, Yingjia Pan, Xiaomeng Guo, Dongfang Li, Guoya Liao, Cui Liu, Xiaoxiang Hu, Guoqing Shao, Zhimin Gu, Lubin Liu, Wen Jiang, Guo Li

**Affiliations:** 1 Xianghu Laboratory, Hangzhou, Zhejiang China; 2 State Key Laboratory of Animal Biotech Breeding, China Agricultural University, Beijing, China; 3 College of Chemical and Biological Engineering, Zhejiang University, Hangzhou, Zhejiang China; 4 ZJU-Hangzhou Global Scientific and Technological Innovation Center, Zhejiang University, Hangzhou, Zhejiang China; 5 Department of Obstetrics and Gynecology, Women and Children’s Hospital of Chongqing Medical University, Chongqing, China; 6 NHC Key Laboratory of Birth Defects and Reproductive Health, Chongqing, China; 7 Department of Obstetrics and Gynecology, Chongqing Health Center for Women and Children, Chongqing, China

**Keywords:** Methylation, Transcriptional regulatory elements

Dear Editor,

Gene expression is regulated at multiple levels, including transcription, RNA processing, and translation^1^. Conventional gene-editing technologies such as CRISPR-Cas9^2^ and DNA base editors^3^ induce permanent genomic alterations through unpredictable DNA repair pathways. Cas13-based systems and derived RNA editors^4^ can alter transcript abundance and introduce off-target RNA effects. Although CRISPR-based transcriptional activators and repressors have enabled precise control at the DNA level^5^, direct and programmable manipulation of protein output downstream of transcription remains limited. In particular, strategies that selectively enhance endogenous protein production without altering genomic sequences or RNA nucleotide composition are still lacking, despite the central role of post-transcriptional regulation in shaping cellular phenotypes and disease states.

Post-transcriptional regulation of mRNA translation represents a critical yet underexploited layer of gene expression control. Among known mechanisms, N^6^-methyladenosine (m^6^A) modification^6^ functions as a pervasive and reversible signal that modulates mRNA fate through the recruitment of reader proteins. Notably, the cytoplasmic m^6^A reader YTHDF1 enhances translation efficiency by facilitating ribosome engagement and translation initiation^7^. These findings suggest that translational output can be selectively amplified through targeted engagement of endogenous RNA regulatory pathways, raising the possibility that protein expression may be programmably modulated at the translational level rather than through transcriptional activation or sequence editing.

Here, we sought to establish a programmable strategy for selectively enhancing endogenous protein expression by directly engaging translation-regulatory machinery. To this end, we leveraged RNA-targeting CRISPR systems to guide an m^6^A-dependent translational reader to specific mRNA transcripts, thereby enabling guide RNA-directed modulation of protein output without inducing RNA cleavage or sequence modification. This approach defines a translation-level regulatory framework that operates downstream of transcription and independently of nucleotide editing.

To address these challenges, we designed CRISPR-mediated Translation Enhancement (CRISPR-TE), which selectively engages endogenous mRNA transcripts without inducing RNA cleavage or sequence modification. Catalytically inactive Cas13j or its compact variant^8^ was fused to the m^6^A reader protein YTHDF1, enabling guide RNA-directed recruitment of translational machinery to target transcripts (Fig. 1a, b; Supplementary Fig. S1a–d and Table S1). Unlike canonical CRISPR-based transcriptional regulators or RNA editors, this strategy modulates protein expression at the translational level rather than through transcriptional or RNA-editing mechanisms.

**Fig. 1:**
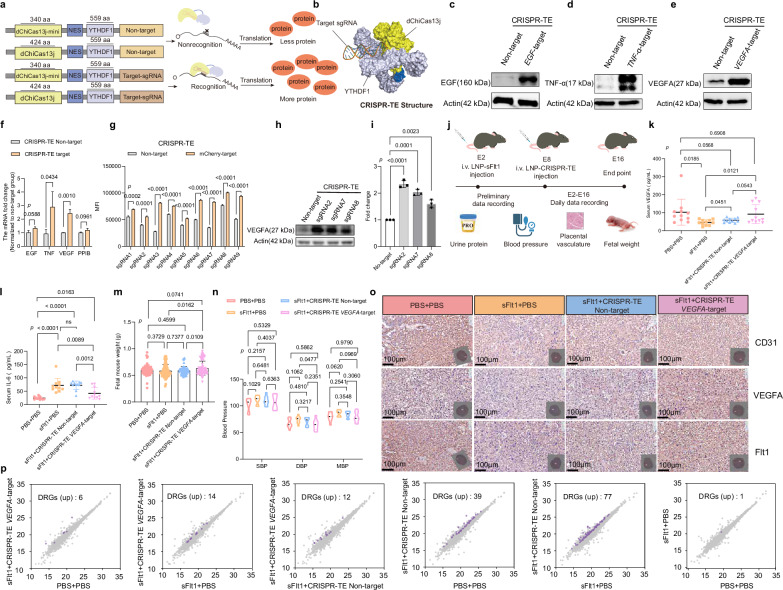
CRISPR-guided epitranscriptomic enhancement of *Vegfa* mRNA translation enables selective protein upregulation and therapeutic rescue in preeclampsia. **a** Schematic of *Vegfa*-targeting CRISPR-TE constructs, including full-length dChiCas13j and compact dChiCas13j-mini fused to YTHDF1. **b** AlphaFold2-predicted structure of the CRISPR-TE effector in complex with sgRNA and target RNA. **c**–**e** Western blot analysis of endogenous EGF (**c**), TNF (**d**) and VEGFA (**e**) protein levels in HEK293T cells transfected with CRISPR-TE and the indicated high-activity sgRNAs (*n* = 3 per group). **f** RIP-qPCR analysis using anti-FLAG antibody showing specific association of CRISPR-TE with target transcripts. *EGF*, *TNF*, *VEGFA* and *PPIB* mRNA abundance was measured by RT-qPCR and normalized to non-targeting controls (*n* = 3 per group). **g** Flow cytometry analysis of mCherry-VEGFA reporter expression in N2a cells expressing CRISPR-TE with individual *Vegfa*-targeting sgRNAs (*n* = 3 per group). **h** Western blot analysis of endogenous VEGFA protein levels in N2a cells transfected with CRISPR-TE and selected *Vegfa*-sgRNAs (*n* = 3 per group). **i** Quantification of VEGFA protein levels in **h**, normalized to β-actin (*n* = 3 per group). **j** Experimental design of the preeclampsia mouse model. Pregnant mice received sFlt1-LNP or PBS at E2, followed by *Vegfa*-sgRNA7 CRISPR-TE-LNP, non-targeting CRISPR-TE-LNP or PBS at E8 (*n* = 15 per group). **k**, **l** Serum VEGFA and IL-6 levels at E16 measured by ELISA (*n* = 11 per group). **m** Fetal body weight at E16 (*n* = 79 in PBS + PBS group, *n* = 78 in sFlt1+PBS group, *n* = 81 in sFlt1+CRISPR-TE Non-target group, *n* = 75 in sFlt1+CRISPR-TE VEGFA-target group). **n** Arterial blood pressure at E14 (*n* = 8 per group). SBP systolic blood pressure, DBP diastolic blood pressure, MBP mean blood pressure. **o** Representative immunohistochemistry staining of placental sections at E16 for CD31, VEGFA and sFlt1 in PBS-treated controls, sFlt1-induced preeclampsia mice, non-targeting CRISPR-TE-treated mice, and *Vegfa*-targeting CRISPR-TE-treated mice. Scale bars, 100 μm (*n* = 3 per group). **p** Quantitative proteomic analysis of placental tissues showing differentially upregulated proteins in the *Vegfa*-targeting CRISPR-TE group relative to PBS-treated controls, sFlt1-induced preeclampsia controls and non-targeting CRISPR-TE controls (*n* = 3 per group). Data are mean ± SEM. Statistical significance was determined by two-tailed Student’s *t*-test.

We next examined whether CRISPR-TE-mediated translation enhancement is guide-dependent. We designed 11 single-guide RNAs (sgRNAs) targeting tumor necrosis factor (*TNF*), vascular endothelial growth factor A (*VEGFA*), and epidermal growth factor (*EGF*) and evaluated their activities using an mCherry reporter assay carrying sgRNA target sequences downstream of the coding region (Supplementary Fig. S1e). An all-in-one vector co-expressing dCas13j-YTHDF1 and the corresponding sgRNA was transfected with the reporter into HEK293T cells, resulting in increased mCherry fluorescence at 72 h (Supplementary Fig. S1f–k). Notably, CRISPR-TE mediated substantial fluorescence enhancement across multiple sgRNAs: by 113%, 102%, and 92% for *TNF*-targeting sgRNAs 1–3. Relative to non-targeting controls, CRISPR-TE increased mCherry reporter output by 115%, 11%, and 96% for *EGF*-targeting sgRNAs 1, 3, and 4, respectively, whereas *EGF*-targeting sgRNA 2 produced a modest reduction, suggesting context-dependent effects. For *VEGFA*-targeting sgRNAs 1–4, reporter expression increased by 94%, 91%, 106%, and 98%, respectively (all *P* < 0.05) (Supplementary Fig. S1f–h). We next validated the activity of CRISPR-TE at endogenous loci using the top-performing sgRNAs targeting *TNF*, *EGF* and *VEGFA*. Western blot analysis showed marked upregulation of the corresponding proteins relative to the non-targeting control (Fig. 1c–e). To examine whether CRISPR-TE also affected RNA abundance, we performed reverse transcription quantitative PCR (RT-qPCR) following targeting of endogenous *TNF*, *EGF*, *VEGFA*, and peptidylprolyl isomerase B (*PPIB*) transcripts. Transcript levels were only modestly altered, with slight increases observed for *EGF* and *TNF* and slight decreases for *VEGFA* and *PPIB*, indicating that the effect of CRISPR-TE on RNA stability is limited and context dependent (Supplementary Fig. S1l). We further performed RNA immunoprecipitation (RIP) assays to determine whether the CRISPR-TE fusion protein associates with endogenous transcripts in an sgRNA-directed manner. Following anti-Flag immunoprecipitation, target mRNAs were selectively enriched relative to the non-targeting control, supporting specific recognition of sgRNA-directed endogenous transcripts by CRISPR-TE (Fig. 1f; Supplementary Fig. S1m). To assess the contribution of the m^6^A-binding, we generated a truncated CRISPR-TE variant by fusing catalytically inactive Cas13j to an m^6^A-binding-deficient YTHDF1 mutant. This mutant CRISPR-TE showed substantially reduced translational enhancement on mCherry reporters carrying *EGF*, *VEGFA*, and *PPIB* target sequences, whereas its activity on the *TNF* reporter was comparable to that of the full-length CRISPR-TE (Supplementary Fig. S1n). This target-dependent difference may reflect the limited dynamic range of the *TNF* reporter or differences in guide RNA-target binding properties across reporter contexts. These data indicate that CRISPR-TE enhances protein expression from target mRNAs in a sequence-specific manner, with the magnitude of activation influenced by sgRNA design and supported in part by the m^6^A-binding function of YTHDF1^9^. Because CRISPR-TE acts at the RNA level without cleavage or nucleotide alteration, it avoids permanent genomic modification and provides a potentially reversible means of enhancing endogenous protein output. The system’s compact architecture, together with its ability to significantly elevate translation output, positions CRISPR-TE as a versatile and promising platform for RNA-level therapeutic editing.

Preeclampsia, a leading cause of maternal-fetal morbidity worldwide, is characterized by hypertension, proteinuria, and endothelial dysfunction, primarily driven by placental insufficiency due to impaired VEGFA signaling^10^ (Supplementary Fig. S2a). VEGFA is essential for placental angiogenesis, and its downregulation often triggered by elevated soluble fms-like tyrosine kinase 1 (sFlt1) plays a central role in disease progression^11^. Currently, no therapies target the underlying placental dysfunction. Existing strategies, such as recombinant VEGFA supplementation, viral-mediated gene overexpression, siRNA-based *sFlt1* knockdown, and CRISPR-Cas gene editing, face major translational hurdles, including poor delivery efficiency, short duration of action, virus-mediated immunogenicity, and risks of genomic alteration. To address this limitation, we tested whether CRISPR-TE could enhance VEGFA protein expression through RNA targeting without introducing permanent genetic changes. We designed 9 sgRNAs targeting mouse *Vegfa* mRNA and constructed corresponding mCherry reporter constructs to identify more effective CRISPR-TE guides for enhancing VEGFA protein expression. When tested in mouse neuroblastoma Neuro-2a (N2a) cells, the full-length dChiCas13j version mediated a stronger upregulation, ranging from 25% to 194% (Fig. 1g; Supplementary Fig. S2b). In comparison, the compact CRISPR-TE increased mCherry fluorescence by 10%–53% across sgRNAs (Supplementary Fig. S2c, d).

These results confirm that CRISPR-TE effectively enhances VEGFA expression in a sgRNA-dependent manner, with the full-length editor exhibiting greater potency. To validate CRISPR-TE’s ability to modulate endogenous VEGFA expression, we selected three high-performing sgRNAs (sgRNAs 2, 7, and 8) and transfected the corresponding CRISPR-TE constructs into N2a cells. Western blot analysis of FACS-sorted mCherry-positive cells further showed that the compact dChiCas13j-mini variant robustly increased VEGFA protein levels by up to 45% (Supplementary Fig. S2e, f), while the full-length dChiCas13j version mediated a stronger upregulation, ranging from 60% to 134% (Fig. 1h, i). To assess the specificity of translational regulation, we performed transcriptomic and quantitative proteomic profiling in cells expressing *Vegfa*-targeting or non-targeting guide RNAs. In RNA-seq analysis, only 7 genes were upregulated in the *Vegfa*-sgRNA7 group compared to the non-targeting group, whereas 76 and 86 genes were upregulated in the *Vegfa*-sgRNA7 and non-targeting control groups, respectively, relative to wild-type (WT) groups (Supplementary Fig. S2g and Table S2). Similarly, quantitative mass spectrometry detected merely 6 upregulated proteins in *Vegfa*-sgRNA7 vs WT, with only 3 protein expression changes between *Vegfa*-sgRNA7 and the non-targeting control (Supplementary Fig. S2h and Table S3). The concordance between transcriptomic and proteomic datasets indicates that CRISPR-guided translational enhancement does not globally rewire gene expression programs or induce widespread translational dysregulation. Instead, protein upregulation is highly selective and confined to targeted transcripts, underscoring the precision of translational epitranscriptomic regulation.

We next assessed whether CRISPR-TE-mediated translational enhancement of *Vegfa* mRNA could restore placental angiogenic signaling and alleviate disease manifestations in an established sFlt1-induced murine model of preeclampsia. Pregnant mice received placenta-tropic lipid nanoparticles (LNPs) encoding sFlt1 at embryonic day 2 (E2) to induce preeclampsia-like pathology, followed at E8 by treatment with LNPs delivering CRISPR-TE paired with a *Vegfa*-targeting sgRNA, a non-targeting sgRNA, or vehicle control. LNPs functionalized with a chondroitin sulfate A-binding peptide enabled efficient placenta-specific delivery without detectable off-target accumulation in other maternal organs^12^. Longitudinal physiological monitoring was performed from E2 to E16, with endpoint analyses of serum, placental, and fetal parameters (Fig. 1j; Supplementary Fig. S3a). CRISPR-TE treatment significantly restored circulating VEGFA levels relative to sFlt1-exposed controls, approaching concentrations observed in healthy pregnancies (Fig. 1k). This restoration occurred without a reduction in circulating sFlt1, indicating that the therapeutic effect was driven by enhanced VEGFA expression rather than suppression of the pathogenic factor. Unexpectedly, serum sFlt1 levels at E16 were lower in the sFlt1-LNP group than in PBS controls, possibly because exogenous sFlt1 had been cleared by the sampling time point and had suppressed endogenous placental sFlt1 production through feedback regulation (Supplementary Fig. S3b). Consistent with improved angiogenic signaling, CRISPR-TE treatment markedly reduced systemic inflammation, as evidenced by decreased serum IL-6 and TNF-α levels compared with disease and non-targeting controls (Fig. 1l; Supplementary Fig. S3c). Despite comparable maternal body weight trajectories across all groups (Supplementary Fig. S3d), fetal growth was improved in the CRISPR-TE-treated group, with increased fetal weights relative to sFlt1-exposed controls (Fig. 1m). CRISPR-TE-treated mice exhibited normalization of proteinuria over the course of pregnancy (Supplementary Fig. S3e) and a significant reduction in arterial blood pressure, while heart rate remained unchanged (Fig. 1n; Supplementary Fig. S3f). These results demonstrate that placenta-targeted CRISPR-TE-mediated enhancement of VEGFA expression effectively rescues angiogenic insufficiency and ameliorates key pathological features of preeclampsia in vivo.

To determine whether CRISPR-TE-mediated *Vegfa* mRNA translation enhancement restores placental angiogenesis in vivo, we analyzed placental vascularization at E16 using immunohistochemical staining for CD31, VEGFA, and sFlt1 across four experimental groups: healthy controls (PBS), sFlt1-induced preeclampsia, non-targeting CRISPR-TE, and *Vegfa*-targeting CRISPR-TE (Fig. 1o). In sFlt1-exposed placentas, CD31-positive vascular areas were markedly reduced, indicating impaired placental angiogenesis, whereas treatment with *Vegfa*-targeting CRISPR-TE robustly restored CD31 staining to levels comparable with healthy controls. In contrast, non-targeting CRISPR-TE produced only a partial recovery of vascular density. VEGFA immunoreactivity closely paralleled the CD31 staining pattern, with strong VEGFA signals observed in both healthy and *Vegfa*-targeting CRISPR-TE-treated placentas, while sFlt1-induced placentas exhibited substantially reduced VEGFA expression. Importantly, sFlt1 protein levels remained elevated in both targeting and non-targeting CRISPR-TE groups, indicating that the observed angiogenic rescue was driven by enhanced VEGFA expression rather than suppression of the pathogenic factor (Supplementary Fig. S3g). Consistent with the immunohistochemical findings, western blot analysis of placental lysates confirmed a pronounced increase in VEGFA protein levels in the *Vegfa*-targeting CRISPR-TE group relative to sFlt1 and non-targeting controls, whereas sFlt1 expression remained unchanged (Supplementary Fig. S3h–j).

To comprehensively assess the in vivo specificity of CRISPR-TE, we performed quantitative proteomic profiling of placental tissues from all experimental groups. Compared with PBS-treated controls, placentas from the *Vegfa*-targeting CRISPR-TE group exhibited only a limited number of upregulated proteins, indicating minimal proteome-wide perturbation (Fig. 1p). In contrast, substantially larger numbers of differentially regulated proteins were observed in the non-targeting CRISPR-TE and sFlt1-only groups, consistent with broader, non-specific proteomic alterations associated with disease induction or untargeted interventions. These results demonstrate that *Vegfa*-targeted CRISPR-TE achieves highly selective protein modulation within the placenta.

To further evaluate potential systemic off-target effects, we examined VEGFA and sFlt1 protein levels across multiple maternal organs, including heart, liver, spleen, lung, and kidney. In the heart and liver, VEGFA and sFlt1 expression levels remained comparable across all experimental groups, indicating negligible off-target modulation in these tissues (Supplementary Fig. S4a–d and Table S4). Similarly, splenic VEGFA and sFlt1 levels showed no significant differences following CRISPR-TE treatment (Supplementary Fig. S4a, b, e). In lung and kidney tissues, modest group-specific variations in VEGFA or sFlt1 expression were observed; however, these changes were not associated with consistent upregulation patterns in the *Vegfa*-targeting CRISPR-TE group and remained within physiological ranges (Supplementary Fig. S4a, b, f, g). Collectively, these placental proteomic and multi-organ analyses indicate that CRISPR-TE-mediated VEGFA activation is predominantly restricted to the placenta, with minimal systemic off-target effects.

Several gene-based strategies for preeclampsia target angiogenic imbalance, including siRNA-mediated suppression of placental *sFlt1*, VEGFA delivery, and miRNA-based modulation of the sFlt1-VEGF axis^13–15^. However, because VEGFA is a central growth factor, non-specific overexpression may introduce unpredictable long-term risks. By contrast, CRISPR-TE selectively enhances translation of endogenous target transcripts with limited transcriptomic and proteomic perturbation. Unlike DNA editing, CRISPR-TE does not introduce permanent genetic alterations, a feature that may be particularly advantageous for treating preeclampsia.

Preeclampsia is a multifactorial disorder arising from the convergence of several pathological processes. Impaired decidualization, abnormal cytotrophoblast invasion, endothelial dysfunction, defective spiral artery remodeling, and excessive release of anti-angiogenic factors, particularly sFlt1, into the maternal circulation all contribute to disease pathogenesis^10^. Excess sFlt1 sequesters VEGF, leading to a substantial reduction in bioavailable VEGF, which is considered a major cause of the maternal vascular manifestations of preeclampsia. This has prompted growing interest in VEGF-centered therapeutic strategies. We therefore established an LNP-sFlt1 mouse model to assess the efficacy and safety of CRISPR-TE-mediated enhancement of *Vegfa* mRNA translation. Although this model does not fully recapitulate the etiology of preeclampsia, it provides a tractable system for therapeutic evaluation and offers insight into the treatment of maternal vascular symptoms associated with the disease.

In summary, we introduce CRISPR-TE as a programmable epitranscriptomic platform that enables selective enhancement of protein expression through translation-level regulation rather than transcriptional control or sequence alteration. By coupling nuclease-inactive Cas13j effectors with the endogenous m^6^A reader YTHDF1, CRISPR-TE harnesses an endogenous RNA modification-dependent mechanism to recruit translational machinery to target mRNAs in a guide RNA-directed manner. This strategy fundamentally differs from existing genome editing, RNA cleavage, or base editing approaches by avoiding permanent genetic changes, RNA strand breaks, or direct modification of nucleotide sequences. Across multiple endogenous targets, CRISPR-TE achieves robust and tunable protein upregulation with minimal transcriptome- and proteome-wide perturbations, underscoring its high specificity. Importantly, targeted delivery of CRISPR-TE to the placenta restores VEGFA expression in an sFlt1-driven mouse model of preeclampsia, leading to recovery of placental angiogenesis and systemic physiological improvement without detectable off-target effects in maternal organs. These findings position translational epitranscriptomic regulation as a distinct and programmable layer of gene control, expanding the conceptual landscape of CRISPR-based technologies beyond transcriptional and sequence editing.

## Supplementary information

Supplementary information

Supplementary Table 1

Supplementary Table 2

Supplementary Table 3

Supplementary Table 4

## References

[1] Delaunay, S., Helm, M. & Frye, M. *Nat. Rev. Genet.* **25**, 104–122 (2024). 37714958 10.1038/s41576-023-00645-2

[2] Jinek, M. et al. *Science* **337**, 816–821 (2012). 22745249 10.1126/science.1225829 PMC6286148

[3] Komor, A. C., Kim, Y. B., Packer, M. S., Zuris, J. A. & Liu, D. R. *Nature* **533**, 420–424 (2016). 27096365 10.1038/nature17946 PMC4873371

[4] Cox, D. B. T. et al. *Science* **358**, 1019–1027 (2017). 29070703 10.1126/science.aaq0180 PMC5793859

[5] Cappelluti, M. A. et al. *Nature* **627**, 416–423 (2024). 38418872 10.1038/s41586-024-07087-8 PMC10937395

[6] Liu, J. et al. *Science* **367**, 580–586 (2020). 31949099 10.1126/science.aay6018 PMC7213019

[7] Wang, X. et al. *Cell* **161**, 1388–1399 (2015). 26046440 10.1016/j.cell.2015.05.014 PMC4825696

[8] Li, G. et al. *Nat. Chem. Biol.* **21**, 280–290 (2025). 39300230 10.1038/s41589-024-01729-8

[9] Rauch, S., He, C. & Dickinson, B. C. *J. Am. Chem. Soc.* **140**, 11974–11981 (2018). 30183280 10.1021/jacs.8b05012 PMC6436614

[10] Mol, B. W. J. et al. *Lancet* **387**, 999–1011 (2016). 26342729 10.1016/S0140-6736(15)00070-7

[11] Levine, R. J. et al. *N. Engl. J. Med.* **350**, 672–683 (2004). 14764923 10.1056/NEJMoa031884

[12] Zhang, B. et al. *Theranostics* **8**, 2765–2781 (2018). 29774074 10.7150/thno.22904 PMC5957008

[13] Turanov, A. A. et al. *Nat. Biotechnol.* **36**, 1164–1173 (2018).

[14] Swingle, K. L. et al. *J. Am. Chem. Soc.* **145**, 4691–4706 (2023). 36789893 10.1021/jacs.2c12893 PMC9992266

[15] Beards, F., Jones, L. E., Charnock, J., Forbes, K. & Harris, L. K. *Theranostics* **7**, 2940–2955 (2017). 28824727 10.7150/thno.18845 PMC5562227

