# Supplementary material for: Programmable enhancement of endogenous mRNA translation through CRISPR-guided epitranscriptomic regulation
Source: Cell Discov. 2026 Jun 24;12:45. doi: 10.1038/s41421-026-00903-7 (PMC13294356; doi:10.1038/s41421-026-00903-7)
Supplement: Supplementary file 1 — Supplementary information [file 41421_2026_903_MOESM1_ESM.pdf]

Supplemental materials

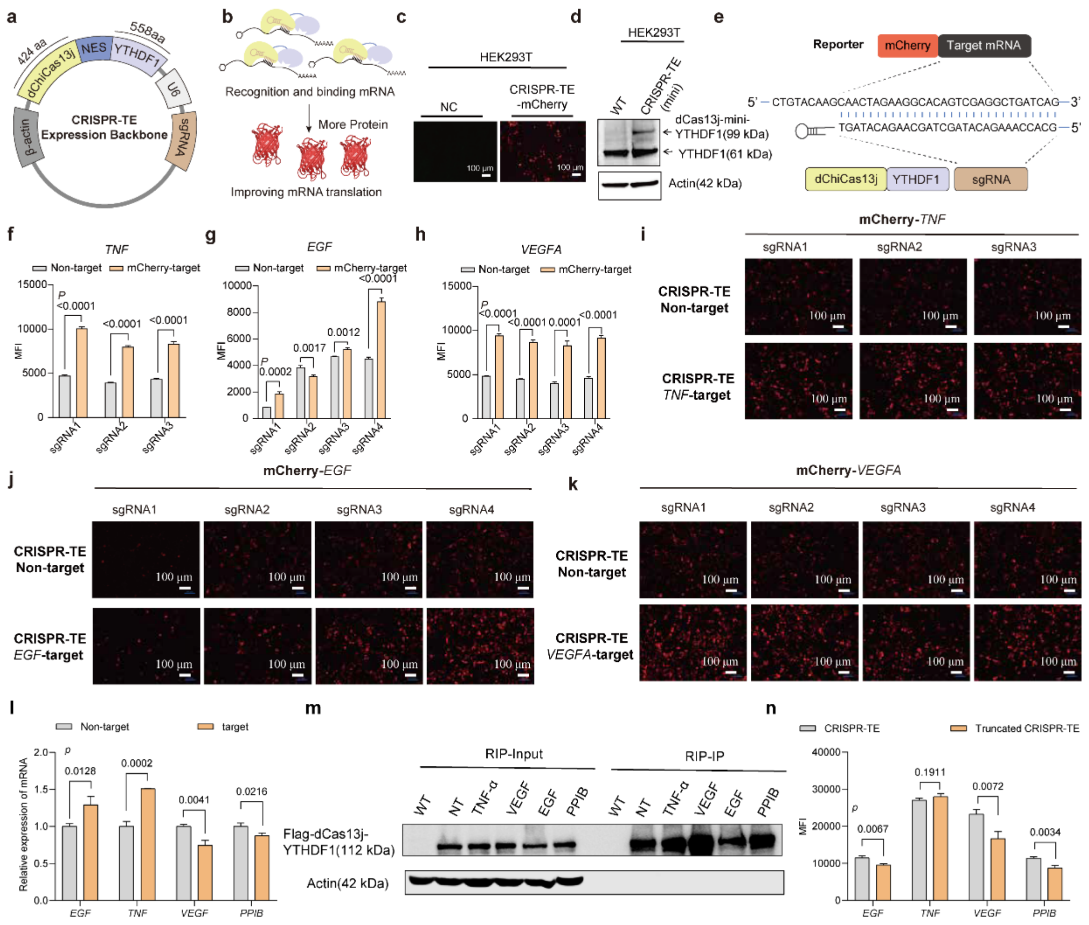

Supplementary Fig. S1 Design, expression, and *in vitro* validation of CRISPR-TE-mediated translation enhancement. a. Schematic diagram illustrating guide RNA-directed engagement of endogenous mRNA translation machinery through epitranscriptomic regulation. CRISPR-TE, the β-actin promoter drives expression of a fusion protein consisting of catalytically inactive dChiCas13j (424 aa) or its compact mini variant (340 aa), fused to the m6A reader protein YTHDF1, together with a U6 promoter-driven sgRNA cassette. **b.** Illustration of the proposed mechanism of action, whereby CRISPR-TE binding to target mRNAs enhances translation efficiency, resulting in increased protein production. **c.** Fluorescence microscopy images of HEK293T cells co-transfected with CRISPR-TE constructs and mCherry-based reporter. Scale bars, 100 μm. **d.** Western blot analysis of YTHDF1

fusion protein expression in HEK293T cells transfected with CRISPR-TE constructs. β-actin was used as a loading control. **e.** Schematic diagram of guide RNA-directed translation enhancement by CRISPR-TE. The guide RNA directs the dCas13j-YTHDF1 combined with target mRNA sequences, enabling YTHDF1-mediated recruitment of translational machinery and increased protein output. **f-h.** Flow cytometry analysis of fluorescence level in HEK293T cells expressing CRISPR-TE and sgRNAs targeting mcherry-based reporters of TNF (f), EGF (g), or VEGFA (h). Data are shown as mean fluorescence intensity (MFI) and represent mean ± s.e.m. from three independent biological replicates. Statistical significance was determined relative to non-targeting controls (two-tailed Student's t-test; exact *P* values indicated). **i-k.** Fluorescence microscopy of HEK293T cells co-transfected with mCherry reporters and dChiCas13j-based CRISPR-TE systems by targeting TNF reporters with sgRNA1-3 (i), EGFA with sgRNA1-4 (j), and VEGFA with sgRNA1-3 (k), respectively. Scale bars: 100 μm. **l.** RT-qPCR analysis of mRNA level in HEK293T cells expressing CRISPR-TE and sgRNAs targeting endogenous EGF, TNF, VEGF and PPIB mRNA, respectively. Data are normalized to non-target control and represent mean ± s.e.m. from three independent biological replicates. Statistical significance was determined relative to non-targeting controls; *P* values were determined by two-tailed Student's t-test. **m.** Western blot analysis of input samples and IP samples using anti-Flag and anti-actin antibody to confirm the presence of Flag-fused CRISPR-TE in IP materials after immunoprecipitation. **n.** Flow cytometry analysis of mcherry fluorescence in HEK293T cells mediated by CRISPR-TE or truncated CRISPR-TE without m6A binding domain when targeting mcherry-based reporters of EGF, TNF, VEGF and PPIB. Data are shown as mean fluorescence intensity (MFI) and represent mean ± s.e.m. from three independent experiments. Statistical significance was determined relative to non-targeting controls (two-tailed Student's t-test; exact *P* values indicated).

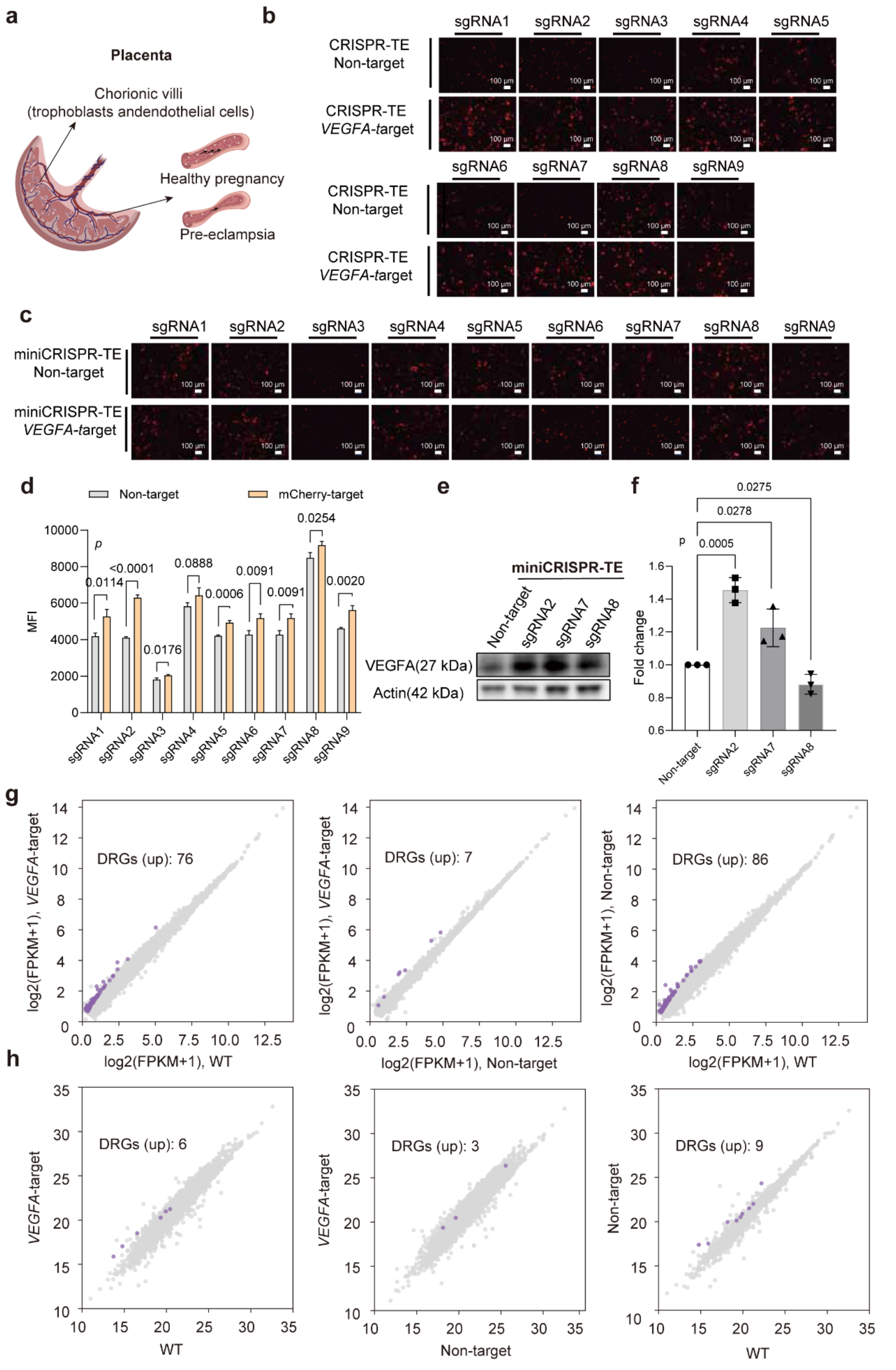

Supplementary Fig. S2 sgRNA screening and specificity evaluation of

CRISPR-TE-mediated VEGFA translation enhancement. a. Schematic comparison of placental structure under healthy pregnancy and preeclampsia conditions, highlighting impaired chorionic villi and reduced angiogenesis associated with VEGFA insufficiency. **b.** Fluorescence microscopy of HEK293T cells co-transfected with mCherry-VEGFA reporters and dChiCas13j-based CRISPR-TE paired with sgRNA1-9. dChiCas13j-based CRISPR-TE refers to the CRISPR-TE fusing catalytically dead ChiCas13j to YTHDF1. Scale bars, 100 μm. **c.** Fluorescence microscopy of HEK293T cells co-transfected with mCherry-VEGFA reporters and miniCRISPR-TE paired with sgRNA1-9. miniCRISPR-TE refers to the CRISPR-TE fusing catalytically dead ChiCas13j-mini to YTHDF1. Scale bars, 100 μm. **d.** Flow cytometry quantification of mean fluorescence intensity (MFI) of mCherry-VEGFA reporters in cells treated with dChiCas13j-mini-based CRISPR-TE ($P < 0.05$; mean ± s.e.m., n = 3). **e.** Western blot detection of endogenous VEGFA in cells transfected with top-performing sgRNAs (2/7/8) and dChiCas13j-mini-based CRISPR-TE. **f.** Densitometric analysis of VEGFA protein levels from (e) ($P < 0.05$; mean ± s.e.m., n = 3). β-actin served as loading control. **g.** Transcriptomic profiling by RNA sequencing of stable N2a cell lines expressing CRISPR-TE with VEGFA-targeting sgRNA7 compared with non-targeting and wild-type controls, showing minimal differentially regulated genes (DRGs). **h.** Quantitative proteomic analysis of the same cell lines as in (g), demonstrating highly selective VEGFA upregulation with limited off-target protein expression changes. Data represent mean ± s.e.m. from three biological replicates.

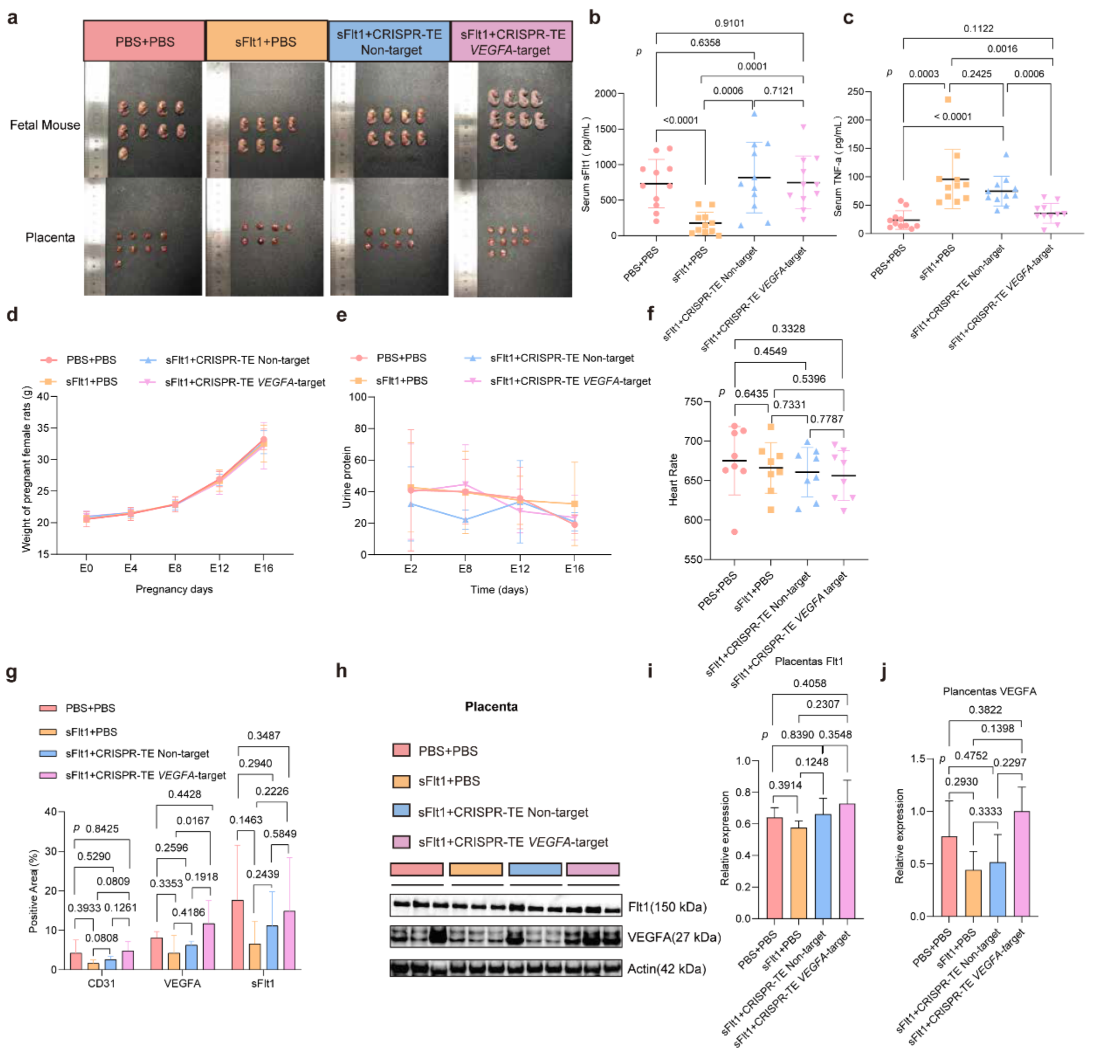

Supplementary Fig. S3 Physiological and placental phenotypic characterization following CRISPR-TE treatment in a mouse model of preeclampsia. a. Representative images of placentas and fetuses at E16 across experimental groups. **b.** Serum sFlt1 concentrations measured by ELISA at E16, showing restoration of circulating VEGFA levels following CRISPR-TE treatment. **c.** Serum TNF-a concentrations measured by ELISA at E16, showing restoration of circulating VEGFA levels following CRISPR-TE treatment. **d.** Longitudinal monitoring of maternal weight throughout gestation ($P < 0.05$; mean ± s.e.m.). **e.** Longitudinal assessment of urinary protein excretion, demonstrating normalization of proteinuria in CRISPR-TE-treated mice. **f.** Maternal heart rate measured at E14, showing no significant differences among groups. **g.** Quantification of CD31-, VEGFA-, and sFlt1-positive staining areas in placental sections

from each group. Data are presented as mean ± s.e.m. from three independent biological replicates; statistical significance was determined by two-tailed Student's t-test, with exact *P* values indicated. **h.** Western blot analysis of VEGFA and sFlt1 protein levels in placental lysates from the indicated groups, with β-actin serving as a loading control. **i-j.** Western blot analysis of VEGF and sFlt1 protein expression in placental from all treatment groups.

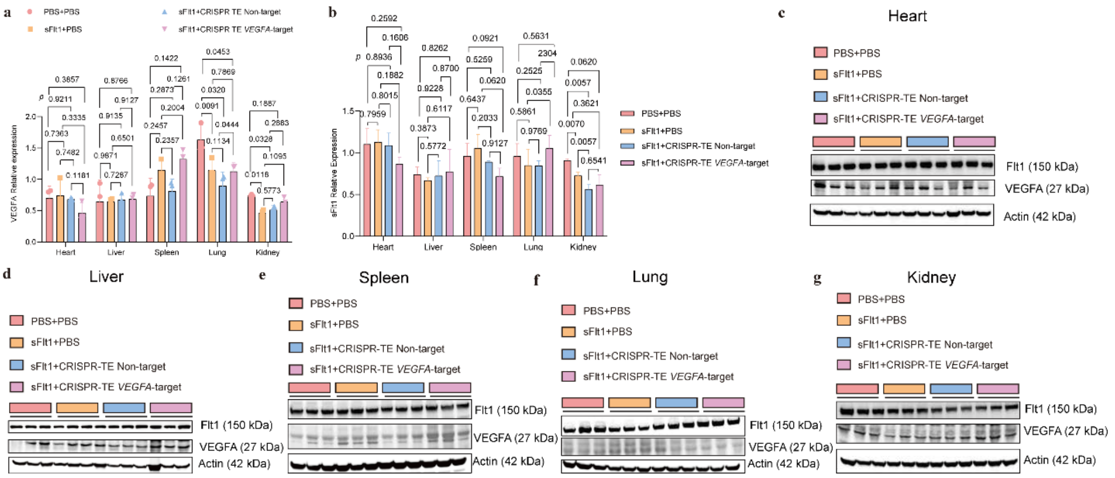

Supplementary Fig. S4 Minimal systemic off-target effects of VEGFA-targeting CRISPR-TE in maternal organs. a. Western blot analysis of VEGFA protein levels in maternal organs, including heart, liver, spleen, lung, and kidney, collected at embryonic day 16 from the indicated experimental groups: PBS-treated healthy controls, sFlt1-induced preeclampsia, sFlt1 plus non-targeting CRISPR-TE, and sFlt1 plus VEGFA-targeting CRISPR-TE. Quantification is shown as mean ± s.e.m. from three biological replicates per group; β-actin was used as a loading control. Exact *P* values are indicated in the panels. **b.** Western blot analysis of sFlt1 protein levels in maternal organs, including heart, liver, spleen, lung, and kidney, collected at embryonic day 16 from the indicated experimental groups: PBS-treated healthy controls, sFlt1-induced preeclampsia, sFlt1 plus non-targeting CRISPR-TE, and sFlt1 plus VEGFA-targeting CRISPR-TE. Quantification is shown as mean ± s.e.m. from three biological replicates per group; β-actin was used as a loading control. Exact *P* values are indicated in the panels. **c-g.** Representative of sFlt1 and VEGFA expression by western blot in heart, liver, spleen, lung, and kidney tissues among CRISPR-TE paired with sgRNA7-LNP (treatment), CRISPR-TE paired with non-target-LNP (control), PBS (healthy control) experimental groups, respectively. β-actin served as loading control for

normalization.

Supplementary Tables

Supplementary Table S1 The sequences of CRISPR-TE and sgRNAs used in this study.

Supplementary Table S2 The different regulated genes (DRGs) in RNA-seq data of CRISPR-TE expression cells.

Supplementary Table S3 The different regulated genes (DRGs) in proteomics data of CRISPR-TE expression cells.

Supplementary Table S4 The different regulated genes (DRGs) in proteomics data of CRISPR-TE expression tissues.

Materials and Methods

Cell Culture and Transfection

HEK293T and Neuro-2a (N2a) cells were cultured in Dulbecco's Modified Eagle Medium (DMEM; Gibco) supplemented with 10% fetal bovine serum (FBS; Excell) and 1% penicillin-streptomycin (Gibco) at 37°C in a 5% CO_2 atmosphere. For transfection, cells were seeded in 24-well plates at a density of 5×10^4 cells per well and allowed to adhere for 18 hours. Transfection was performed using EZ Trans reagent (Shanghai Life iLab Biotech) with 1 μg plasmid DNA per well according to the manufacturer's protocol. Cells were harvested 48 hours post-transfection for subsequent analysis.

Plasmid Construction

The CRISPR-TE effector constructs encoding dChiCas13j-YTHDF1 and dChiCas13j-mini-YTHDF1 fusions were cloned into a mammalian expression vector under the control of the chicken β-actin promoter (Genscript). Catalytic inactivation of both dChiCas13j and dChiCas13j-mini variants was achieved through PCR-based mutagenesis of RRxxxH/RHxxxH motifs. For sgRNA design: Target-specific

sequences were designed and cloned into all-in-one plasmids using Golden Gate Assembly with BbsI-linearized vectors. All oligonucleotide and sgRNA sequences are provided in Supplementary Tables.

RNA Sequencing (RNA-seq)

Total RNA was isolated from N2a cells and mouse placental tissues using TRIzol reagent (Vazyme) according to the manufacturer's instructions. RNA integrity was confirmed by agarose gel electrophoresis, and concentration was determined using a NanoDrop spectrophotometer. Stranded RNA-seq libraries were constructed from 1 μg of total RNA using the Illumina Stranded Total RNA Library Prep Kit with Ribo-Zero Plus rRNA Depletion (Illumina, #20040529), following the standard workflow comprising RNA fragmentation, cDNA synthesis, adapter ligation, and PCR amplification. Library quality was assessed using a Bioanalyzer 2100 (Agilent Technologies), and quantified by qPCR (Kapa Biosystems). Sequencing was performed on an Illumina NextSeq 2000 platform (NextSeq Control Software v1.5.0.42699) to generate 50 million paired-end reads (2×150 bp) per sample, with a Q30 score >80%.

Raw sequencing data were processed using the nf-core RNA-seq pipeline (v3.6), which included quality control (FastQC v0.11.9), adapter trimming (Trim Galore! v0.6.6), and alignment to the GRCm38 mouse reference genome (STAR v2.7.9a). Transcript abundance was quantified using Salmon (v1.6.0) and normalized as transcripts per million (TPM). Differential gene expression analysis was performed using DESeq2 (v1.38.0) with thresholds of |log2 fold change| ≥ 1 and adjusted p-value < 1×10^-5 (Benjamini-Hochberg correction). Data visualization including principal component analysis, MA plots, and volcano plots was generated using Python (v3.10.1) with matplotlib (v3.7.2) and seaborn (v0.13.0) libraries. All analysis scripts were documented in Jupyter notebooks to ensure reproducibility.

Fluorescence Reporter Assays

Cells seeded in 24-well plates were co-transfected with 800 ng of CRISPR-TE expression plasmid and 200 ng of mCherry-Target reporter plasmid per well using the transfection protocol described above. For each experimental condition, triplicate biological replicates were performed. At 48 hours post-transfection, live-cell fluorescence imaging was conducted using a Nikon Eclipse Ti2 inverted microscope equipped with appropriate filters for mCherry detection (excitation/emission: 587/610 nm). For quantitative analysis, parallel samples were processed by flow cytometry using a BD FACS Aria III system (BD Biosciences), where 20,000 single-cell events were recorded per sample with standardized photomultiplier tube voltages and compensation settings. Fluorescence intensity was quantified using ImageJ (v2.14.0) for microscopic images and FlowJo (v10.8.1) for flow cytometry data, with mean fluorescence intensity (MFI) values normalized to untransfected control cells.

Western Blotting

Protein lysates were extracted from cells and tissues using PIRA lysis buffer (X-blot) and clarified by centrifugation (12,000 × g, 10 min). Proteins were separated by SDS-PAGE and transferred to PVDF membranes. After blocking with 5% non-fat milk in TBST (1 h, RT), membranes were incubated overnight at 4°C with primary antibodies against VEGFA (Abcam, # ab46154), sFlt1 (Abcam, # ab32152), and β-actin (Huabio, # ET1702-67) (1:2000 diluted), followed by three 5-min TBST washes. HRP-conjugated goat anti-rabbit IgG (H&L; 1:10,000 diluted) was applied for 1 h at RT. Signals were detected using enhanced chemiluminescence (ECL) and quantified by densitometry (ImageJ v1.53). Three independent biological replicates were performed for each experiment.

Immunohistochemistry (IHC)

Placental tissues were fixed in formalin, paraffin-embedded, and sectioned at 4 μm. Following deparaffinization (xylene) and rehydration (graded ethanol), heat-induced epitope retrieval was performed in 0.01 M citrate buffer (pH 6.0, 95°C,

20 min) using a pressure cooker. Sections were then treated with 3% H_2O_2 (10 min) to quench endogenous peroxidase activity and blocked with 3% non-fat dry milk in TBST (1 h, RT). Primary antibodies against CD31 (Proteintech, #11265-1-AP), VEGFA (Zen-Bioscience, #251622), sFlt1 (Abcam, #ab32152) were applied overnight at 4°C in a humid chamber (Primary antibodies 1:2000 diluted). After three 5-min PBS washes, sections were incubated with HRP-conjugated secondary antibody (1:10,000; Invitrogen A27036) for 1 h at RT. Immunoreactivity was visualized using DAB substrate, with the reaction stopped by distilled water rinsing. Sections were counterstained with Mayer's hematoxylin, dehydrated, and mounted with Permount. Each staining batch included positive and negative controls. Two blinded pathologists evaluated staining patterns using an Olympus BX53 microscope.

Lipid Nanoparticle (LNP) Formulation

Placenta-tropic LNPs were prepared as described (Zhang et al., Theranostics, 2018)[16], encapsulating sFlt1 or CRISPR-TE plasmids (10 μg/dose). Soybean lecithin and 1,2-distearoyl-sn-glycero-3-phosphoethanolamine-N-maleimide (polyethylene glycol 2000) carboxylic acid (DSPE-PEG-COOH) were purchased from Avanti Polar Lipids (Alabama, USA). Poly (lactide-co-glycolide) (PLGA), 1-ethyl-3-(3-dimethylaminopropyl) carbodiimide hydrochloride (EDC), N-hydroxysuccinimide (NHS), methotrexate (MTX) and indocyanine green (ICG) were obtained from Sigma-Aldrich (Missouri, USA). Placental CSA-binding peptide (plCSA-BP, EDVKDINFDTKEKFLAGCLIVSFHEGKC) was purchased from ChinaPeptides Co.,Ltd. (Shanghai, China).

Proteomics

Protein lysates from N2a cells and mouse placental tissues were quantified using the Bradford assay (BSA standard). For mass spectrometry, proteins were reduced (5 mM DTT, 37°C, 30 min), alkylated (15 mM iodoacetamide, dark, 15 min), and digested with trypsin (1:50 w/w, 37°C, overnight). Peptides were desalted (C18

columns), lyophilized, and separated by nano-LC (EASY-nLC system; C18 column, 5-30% acetonitrile gradient over 120 min). MS/MS analysis was performed on an Orbitrap mass spectrometer in data-dependent mode (full MS scan followed by top 10 precursor fragmentation). Raw data were processed with Proteome Discoverer (v2.4) and searched against UniProtKB/Swiss-Prot using these parameters: trypsin/P specificity, fixed carbamidomethylation, variable methionine oxidation, and 1% FDR. Label-free quantification (LFQ) identified differentially expressed proteins (fold change >1.5 or < 0.67; P < 0.05, Student's t-test). Functional enrichment (DAVID, STRING) and protein-protein interaction networks (Cytoscape) were analyzed. Key targets were validated by Western blot: proteins were resolved by SDS-PAGE, transferred to PVDF membranes, and probed with primary antibodies followed by HRP-conjugated secondaries. Chemiluminescent signals were quantified (ImageJ). All experiments included biological triplicates; data are mean ± SD. Differentially expressed proteins (fold change > 1.5 or < 0.67, $P < 0.05$) were analyzed.

Animal Studies

MSLT BIOTECH performed mouse-related experiments with ethics oversight approval (Institutional Animal Care and Use Committee, no. MSLT-2024-0020). All mouse-related experiments adhered to the Regulations on the Administration of Laboratory Animals formulated by the State Science and Technology Commission of the People's Republic of China, the Guiding Opinions on the Treatment of Laboratory Animals issued by the Ministry of Science and Technology of the People's Republic of China and the Measures for the Administration of Laboratory Animals of Zhejiang Province issued by the People's Government of Zhejiang Province. C57BL/6J mice, in their adult phase, were sourced from MSLT BIOTECH. The mice were accommodated in a specific pathogen-free environment and subjected to a 12-h light-dark cycle, with the temperature regulated between 20 and 22 °C and the humidity level maintained at 40-60%. All mice had free access to food and water in standard cages. Pregnant mice were obtained by mating 8-week-old adult female mice

with male mice. The pregnant mice were divided into four groups randomly. The E2 pregnant mice were injected sFlt1 expression vectors through the tail vein with LNP at dose of 10 μg per mouse. Then E8 pregnant mice were injected dCas13jMIni-YTHDF1 expression vectors at dose of 10 μg per mouse. At E16 all pregnant mice were killed, blood samples were collected from the eye socket and tissues were taken. The serum was obtained by centrifuging blood collected through retro-orbital puncture from mice. The levels of VEGFA, sFlt1, IL-6 and TNF-α in mouse serum were measured using ELISA kits (BYabscience).

Statistics

All quantitative data are presented as mean ± standard error of the mean (SEM) derived from a minimum of three biologically independent replicates, where biological independence was defined as experiments performed with distinct cell passages or tissue samples. Statistical analyses were conducted using GraphPad Prism, employing unpaired two-tailed Student's t-tests for pairwise comparisons. Power analysis (G Power v3.1.9.7) confirmed adequate sample sizes ($\beta = 0.8$, $\alpha = 0.05$) to detect effect sizes exceeding 1.5-fold changes across all experimental groups.

Data availability

The RNA-seq data for CRISPR-TE's off-targets detection are available in the National Center for Biotechnology Information (NCBI) Sequence Read Archive (SRA) database under accession code PRJNA1293855. The Proteomics sequencing data for CRISPR-TE-mediated VEGFA proteins expression and off-targets detection are available in the EBI PRIDE (PRoteomics IDEntifications Database) database under accession code PXD067420.
